# Mismatch Negativity and Impaired Social Functioning in Long-Term and in First Episode Schizophrenia Spectrum Psychosis

**DOI:** 10.3389/fpsyt.2020.00544

**Published:** 2020-06-16

**Authors:** Timothy K. Murphy, Sarah M. Haigh, Brian A. Coffman, Dean F. Salisbury

**Affiliations:** Clinical Neurophysiology Research Laboratory, Department of Psychiatry, Western Psychiatric Hospital, University of Pittsburgh School of Medicine, Pittsburgh, PA, United States

**Keywords:** mismatch negativity, schizophrenia, first episode psychosis, social functioning, biomarker

## Abstract

Mismatch negativity (MMN) is elicited by infrequent physical parameter sound changes. MMN to pitch-deviants (pMMN) and duration-deviants (dMMN) are severely reduced in long-term schizophrenia (Sz). Although symptom factors (positive, negative, cognitive) are inconsistently associated with MMN amplitude in Sz, several studies have shown smaller dMMN is associated with impaired social functioning in Sz. MMN is less reduced at the first psychotic episode in the schizophrenia spectrum (FESz). Meta-analyses demonstrate that pMMN is not reduced, while dMMN is moderately impaired. Correlations of pMMN and dMMN with symptom factors in FESz are also equivocal. Associations with social functioning have not been reported. FESz and matched controls (n = 40/group), and Sz and matched controls (n = 50/group) were assessed for baseline and current cognitive functioning, symptoms, and social functioning, and pMMN and dMMN were recorded. Sz showed reductions in pMMN (p = 0.001) and dMMN (p = 0.006) amplitude. By contrast, pMMN (p = 0.27) and dMMN (p = 0.84) were not reduced in FESz. However, FESz showed associations between both MMNs and negative symptoms and social functioning. More impaired MMNs in FESz were associated with increased negative symptoms and impaired social functioning, both current and in the year prior to the emergence of psychosis. These data suggest that the extent of pathological process occurring before first psychosis as reflected in compromised social behavior prior to first break and reduced interpersonal communication and increased alogia at first break is indexed by pMMN and dMMN, putative biomarkers of disease progression sensitive to functional impairment.

## Introduction

Mismatch negativity (MMN) is a neurophysiological response to stimulus deviance typically elicited by infrequent physically deviant tones amongst repeated standard tones. Pitch-deviant MMN (pMMN) and duration-deviant MMN (dMMN) are revealed by subtraction of the averaged event-related potential (ERP) waveform to the standard tone from the deviant ERP waveform (1), and are robustly reduced in long-term schizophrenia (Sz) (2–4). Michie et al. (5) suggested dMMN was a robust intermediate endophenotype for Sz. Initial reports (6) suggested impaired MMN was associated with increased negative symptoms, while later reports failed to replicate those associations and reported associations between smaller MMN and greater positive symptoms (7–9). Recently, a meta-analysis by Erickson et al. (10) suggested that MMN did not reliably correlate with symptom severity in Sz, suggesting no stable MMN-symptom associations.

Despite the large effect sizes for reduction of pMMN and dMMN in Sz (Cohen’s d >1.0) (11), reductions at the first episode of, or during the first hospitalization for, psychosis within the schizophrenia spectrum (FESz) are less robust. Two recent meta-analyses indicated smaller effect sizes for MMN reduction in FESz. Erickson, Ruffle, and Gold (12) reported d = 0.81 in Sz, and d = 0.42 in FESz, collapsing across pMMN and dMMN. Our meta-analysis (13) indicated a negligible effect for pMMN reduction in FESz (d = 0.04), and a small effect for dMMN reduction in FESz (d = 0.47). Because pMMN and dMMN correlate with estimated premorbid IQ (9), analysis including only studies controlling for estimated premorbid IQ revealed an effect size of dMMN reduction in FESz of d = 0.36. Still, the small-to-medium effect size for dMMN reduction at first episode is consistent with its putative role as an intermediate endophenotype.

Although we have not observed pMMN (4, 9, 14) or dMMN (9) reductions in FESz, we have observed pathological associations between brain structure and MMN in FESz. The size of pMMN was associated with the underlying volume of left auditory cortex at first hospitalization, such that individuals with reduced pMMN at first hospitalization had the smallest auditory cortex volume, and both pMMN and left auditory cortex gray matter showed inter-related peri-onset reductions (15). This progressive gray matter loss is greatest in left hemisphere, and in temporal and frontal cortices (16–25). (One exception included progressive loss beginning in parietal cortex, but the subjects were very early onset patients (26)). Ultra-high risk for psychosis subjects who later converted to psychosis had smaller temporal and frontal cortex and progressive loss (27), and progressive loss of left temporal gray matter was greater in genetic high risk converters (28). Thus, progressive brain volume loss is present prior to the emergence of psychosis, and continues during the early disease stage.

Psychosis likely reflects a relatively late stage of the progressive gray matter loss. Psychosis, then, is metaphorically like tremor in Parkinson’s disorder which emerges after ~50–70% of the nigral dopamine neurons have withered (29). Thus, we (14) and others (10) have argued MMN is better conceptualized as a marker of disease progression, sensitive to peri-onset cortical gray matter reduction. Logically, at first episode, some individuals will have smaller MMNs and reduced cortical gray matter due to more severe pre-psychosis disease process. If so, these deficits should also be reflected in differential functional decline prior to the emergence of psychosis.

During the prodromal period, functional impairments arise progressively. Keshavan (personal communication, November 18, 2016) coined the acronym CLAASSIC for this progression, representing deficits first in Cognition and Learning, then in Affect with increased Anxiety, Social deficits, and finally, Subthreshold to Intermittent to Chronic psychotic symptoms. Similarly, Cornblatt et al. (30) coined the acronym CASIS for progressive impairments in Cognitive, Affective, Social, (increased) Isolation, and School/work functioning. In both frameworks, social deficits reflect an intermediate stage of the progressive impairments prior to psychosis. Indeed, among clinical high risk individuals, Cornblatt et al. (31) reported that social and role functioning were more impaired in individuals converting to psychosis than in individuals that did not. We hypothesize deficits in MMN at first psychosis will correlate with impairments in social and role functioning during the prodromal state.

In terms of social functioning and MMN, seminal work by Light and colleagues (32) demonstrated that dMMN was a sensitive index of social functioning in Sz, larger in individuals with better social functioning, a finding since replicated by other groups (33). Recently, structural equation modeling (SEM) indicated that impairments in basic auditory processing reflected partly in dMMN had effects on cognition and negative symptoms, which in turn affected social behavior, suggesting dMMN moderated psychosocial functioning (33). In this study, we specifically utilized the Global Functioning: Social and Global Functioning: Role scales (34). These scales were specifically designed to assess social functioning in the prodromal stage of psychosis, and allow for estimates of the highest and lowest levels of social functioning within the year prior to the transition to psychosis. More specifically, the Social measure addresses the number of friends, the quality of interactions with friends, dating, and family interactions. The Role scale assesses the quality of performance and independence in work, school, and home activities. Higher scores reflect better performance on both scales. We present findings relating reductions in pMMN and dMMN in FESz (within the larger context of within-normal-limits group mean MMN amplitudes) to social functioning and in particular social role functioning in the year prior to first psychosis in the largest sample of FESz to date (n = 40), after first validating the MMN protocols in Sz (n = 50).

## Materials and Methods

### Participants

All Sz-spectrum participants were recruited from Western Psychiatric Hospital (WPH) inpatient and outpatient clinics. Fifty Sz were compared with 50 healthy control participants (HCSz). Forty-one Sz had a diagnosis of Sz (disorganized = 1, paranoid = 14, residual = 9, undifferentiated = 18), and nine schizoaffective disorder (depressed subtype = 5, bipolar subtype = 4). All Sz had at least 5 years of illness duration or were hospitalized a minimum of three times for psychosis. All Sz were taking anti-psychotic medication. Forty FESz were compared with 40 matched controls (HCFE). Eighteen FESz were diagnosed with Sz (paranoid = 12, undifferentiated = 6), 4 with schizoaffective disorder (depressed subtype = 3, bipolar subtype = 1), 10 with psychotic disorder NOS, and 8 with schizophreniform disorder. FESz participated within two months of their first clinical contact for a first episode of psychosis, and had less than 2 months of lifetime antipsychotic medication exposure. Fifteen FESz (35%) were unmedicated.

All subjects had normal hearing (confirmed with audiometry) and a minimum of nine years of schooling. No participant had a history of concussion or head injury with sequelae, alcohol or drug addiction, detoxification in the last five years, or a comorbid neurological disorder that might impact EEG. Participant groups were matched for age, gender, Wechsler Abbreviated Scale of Intelligence (WASI) IQ, and parental socioeconomic status (SES). The four-factor Hollingshead Scale was used to measure SES in participants and in their parents (pSES), and as expected, Sz had lower SES than HCSz (p < 0.001) and FESz were lower than HCFE (p = 0.009), consistent with social and occupational impairment from psychosis (see **Table 1** for demographic measures). All participants provided informed consent. The study was approved by the University of Pittsburgh IRB. Participants were paid.

**Table 1:** Demographic and clinical information.

	Sz	HCSz	p	FESz	HCFE	p
**Age**	35.1 (7.9)	34.9 (10.4)	0.91	23.0 (4.7)	23.6 (4.6)	0.56
**Gender (M/F)**	33/17	32/18	0.83	30/10	28/12	0.62
**SES**	32.0 (13.1)	44.4 (11.1)	**<.001**	30.4 (12.5)	37.3 (10.2)	**0.009**
**Parental SES**	37.3 (14.4)	40.3 (11.6)	0.25	41.2 (13.4)	45.9 (12.5)	0.11
						
**PANSS Total**	68.2 (12.8)			75.4 (15.2)		
**PANSS Positive**	15.2 (5.0)			20.1 (5.4)		
**PANSS Negative**	18.7 (5.4)			17.1 (5.0)		
**PANSS General**	34.3 (5.7)			38.2 (8.3)		
**PANSS TD**	8.3 (3.0)			11.3 (3.2)		
**SAPS Global**	4.0 (2.9)			6.5 (3.5)		
**SANS Global**	11.8 (3.2)			9.4 (4.2)		
**Illness duration**	12.2 (7.4)^a^			17.2 (21.2)^b^		
**DUP**	–			1.6 (2.5)		
**Medication**	783.3 (1273.4)			269.7 (336.9)		

Values are mean (SD). Age in years. Illness duration in years. SES, Socio-Economic Status; PANSS, Positive and Negative Syndrome Scale; SAPS, Scale for the Assessment of Positive Symptoms; SANS, Scale for the Assessment of Negative Symptoms; Illness duration, time since first hospitalization or first contact with psychiatric services; ^a^years ill, ^b^days ill. DUP, Duration of untreated psychosis in years. Medication in chlorpromazine equivalents [oral dosages from Andreasen et al., 2010 (35), depot dosages from Gardner et al., 2010 (36)]. Bolded means statistically significantly different

### Diagnostic Assessments

Psychiatric diagnoses utilized the Structured Clinical Interview for DSM-IV (SCID-P). Symptoms were measured with the Positive and Negative Symptom Scale (PANSS), Scale for Assessment of Positive Symptoms (SAPS), and Scale for Assessment of Negative Symptoms (SANS). All tests were conducted by expert diagnosticians independent from the EEG laboratory (see **Table 1** for clinical measures).

### Neuropsychological and Social Functioning

Participants underwent MATRICS Cognitive Consensus Battery and WASI neuropsychological testing. Social functioning was measured using the Global Assessment of Functioning Scale (GAF), Global Functioning: Social and Role scales (GF), the brief UCSD Performance-based Skills Assessment (UPSA-B), and the Social Functioning Scales (SFS) in clinical samples only (see **Table 2** for neuropsychological test and social functioning scores).

**Table 2:** Neuropsychological and social functioning information.

	Sz	HCSz	p	FESz	HCFE	p
**WASI IQ**	102.9 (12.4)	104.5 (9.5)	0.47	108.4 (13.1)	108.0 (9.4)	0.89
**MATRICS Speed**	38.5 (13.1)	51.7 (10.7)	**<0.001**	45.4 (14.1)	52.3 (7.6)	**0.008**
**MATRICS Att Vig**	41.0 (12.3)	49.4 (8.8)	**<0.001**	42.4 (11.6)	47.6 (8.2)	**0.02**
**MATRICS WM**	40.1 (11.5)	46.0 (8.6)	**0.004**	46.1 (13.8)	45.8 (9.0)	0.89
**MATRICS Verbal**	37.2 (10.5)	49.7 (10.4)	**<0.001**	47.5 (11.0)	50.8 (11.1)	0.18
**MATRICS Visual**	35.9 (11.9)	45.4 (9.1)	**<0.001**	43.4 (11.4)	43.5 (9.5)	0.98
**MATRICS Reason**	44.1 (10.2)	49.3 (9.3)	**0.009**	49.4 (10.2)	50.9 (7.4)	0.46
**MATRICS Soc Cog**	39.2 (12.7)	52.6 (9.3)	**<0.001**	46.4 (11.3)	52.1 (10.1)	**0.02**
**MATRICS Overall**	33.4 (13.3)	48.4 (8.9)	**<0.001**	42.7 (13.3)	48.2 (8.7)	**0.03**
						
**GAF**	44.1 (12.8)			43.5 (11.9)		
**UPSA-B Comm**	6.9 (1.7)			6.8 (1.2)		
**UPSA-B Finance**	8.6 (1.7)			9.0 (1.7)		
**UPSA-B Total**	77.1 (13.9)			79.1 (10.6)		
**GF Role current**	4.8 (2.1)			6.0 (2.3)		
**GF Role lowest**	4.2 (1.9)			5.6 (2.2)		
**GF Role highest**	5.0 (2.0)			7.6 (1.7)		
**GF Social current**	5.2 (1.8)			5.6 (1.9)		
**GF Social lowest**	4.7 (1.8)			5.4 (1.8)		
**GF Social highest**	5.4 (1.8)			7.3 (1.4)		
**SFS Eng/Wth**	9.8 (2.3)			9.7 (2.2)		
**SFS Interpersonal**	7.3 (1.5)			7.3 (1.9)		
**SFS Recreation**	18.8 (5.9)			19.1 (4.8)		
**SFS Occ/Emp**	5.2 (2.6)			8.2 (2.4)		
**SFS Indep/Perf**	26.0 (6.8)			24.0 (6.6)		
**SFS Indep/Comp**	37.0 (2.3)			36.3 (3.7)		
**SFS Prosocial**	15.0 (8.5)			15.8 (7.1)		

Values are mean (SD). WASI, Wechsler Abbreviated Scale of Intelligence; MATRICS, Measurement and Treatment Research to Improve Cognition in Schizophrenia Consensus Cognitive Battery (45); MATRICS Speed, Speed of processing t-score; MATRICS Att Vig, Attention/vigilance t-score; MATRICS WM, Working memory t-score; MATRICS Verbal, Verbal memory and learning t-score; MATRICS Visual, Visual learning and t-percentile score; MATRICS Reason, Reasoning and problem solving t-score; MATRICS Soc Cog, Social cognition t-score; MATRICS Overall, Overall t-score; GAF, Global Assessment of Functioning Scale score; UPSA-B, Brief UCSD Performance-based Skills Assessment; UPSA-B Comm, Communication raw score; UPSA-B Finance, Finance raw score; UPSA-B Total, Total percentile score; GF, Global Functioning Scales; GF Role current/lowest/highest, GF Role scale current (last month)/lowest past year/highest past year; GF Social current/lowest/highest, GF Social scale current (last month)/lowest past year/highest past year (22); SFS, Social Functioning Scales; SFS Eng/Wth, Engagement/Withdrawal raw score; SFS Interpersonal, Interpersonal Communication/Interaction raw score; SFS Recreation, Recreation raw score; SFS Occ/Emp, Occupation/Employment raw score; SFS Indep/Perf, Independence/Performance raw score; SFS Indep/Comp, Independence/Competence raw score; SFS Prosocial, Prosocial raw score. Bolded means statistically significantly different.

### MMN Procedure

Auditory stimuli were presented while EEG was recorded and participants watched a silent video. Stimuli comprised a standard tone (1 kHz, 50-ms duration, 5-ms rise/fall, 80 dB), a pitch deviant (1.2 kHZ, 50-ms duration, 5-ms rise/fall, 80 dB), and a duration deviant (1 kHZ, 100-ms duration, 5-ms rise/fall, 80 dB), presented with a stimulus onset asynchrony of 330 ms. A total of 1,600 tones were presented, including 1,280 standards (80%), 160 pitch deviants (10%), and 160 duration deviants (10%). (Due to time constraints, a subset of 6 FESz, 13 HCFE, 14 Sz, and 16 HCSz participants were tested on only 800 tones, including 640 standards (80%), 80 pitch deviants (10%) and 80 duration deviants (10%). Results did not differ when only the first 800 stimuli were measured for all participants.)

### EEG

EEG was recorded using a custom 72 channel Active2 high impedance system (BioSemi) cap. Sites included 70 10-10 scalp sites including left and right mastoids, the nose tip, and 1 electrode below the right eye. The bandpass was DC to 104 Hz (24 dB/octave roll off) digitized at 512 Hz. EEG was referenced to a common mode sense site (near PO1), with an active “driven-right leg” electrode on the homologous right site.

### MMN Measurement

Off-line processing used BESA (BESA GmbH) where EEG was filtered between 0.5 Hz to remove DC drifts and skin potentials and 20 Hz to remove muscle and other high frequency artifact. Data were visually examined and any channels with excessive noise were interpolated, and ICA was used to remove eye blinks and horizontal eye movements. Next, using BrainVision Analyzer2 (Brain Products GmbH), data were rereferenced to the nosetip, epochs of 350 ms, including a 50-ms prestimulus baseline, were extracted to deviant tones and the standard tones preceding a deviant, baseline correction was applied to each trial, trials exceeding ±50 μV at any scalp site were rejected, and averages constructed for the standard tones preceding a deviant, pitch deviants, and duration deviants. pMMN and dMMN were visualized by subtracting the standard average from the appropriate deviant average. MMN was measured over frontal sites, using FCz peak latency, as average voltage over a 50-ms window (peak ±25 ms).

### Analyses

Group demographics and neuropsychological scores were compared with t-tests and chi-squared tests. MMN analyses utilized repeated-measures ANOVA, with group (Sz, HC) as the between subjects factor, and electrode chain (F, FC, C), and site (left, central, or right) as within subjects factors. The Huynh-Feldt epsilon was used to correct for possible non-sphericity of repeated measures factors containing more than 2 levels. Although source-localization was not a primary aim of this study, CSD topography of pMMN and of dMMN were calculated to indicate consistency with the expected temporal lobe dipole source. Interpolation was done using spherical splines, with the order of splines set to 4, the maximum degree of Legendre polynomials set to 10, and a default lambda of 1e-5. Two-tailed Spearman’s correlations were used to examine relationships between MMN at FCz (where it was largest) and demographic, clinical, neuropsychological, and social functioning items. Raw or t-scores were used for correlations. (Note slight differences in reported p values are due to rounding correlation coefficients to 2 decimal places.) Values are reported as Mean ± SD. Significance was attained at p < .05.

## Results

### Long-Term Sz

#### pMMN

**Figure 1:**
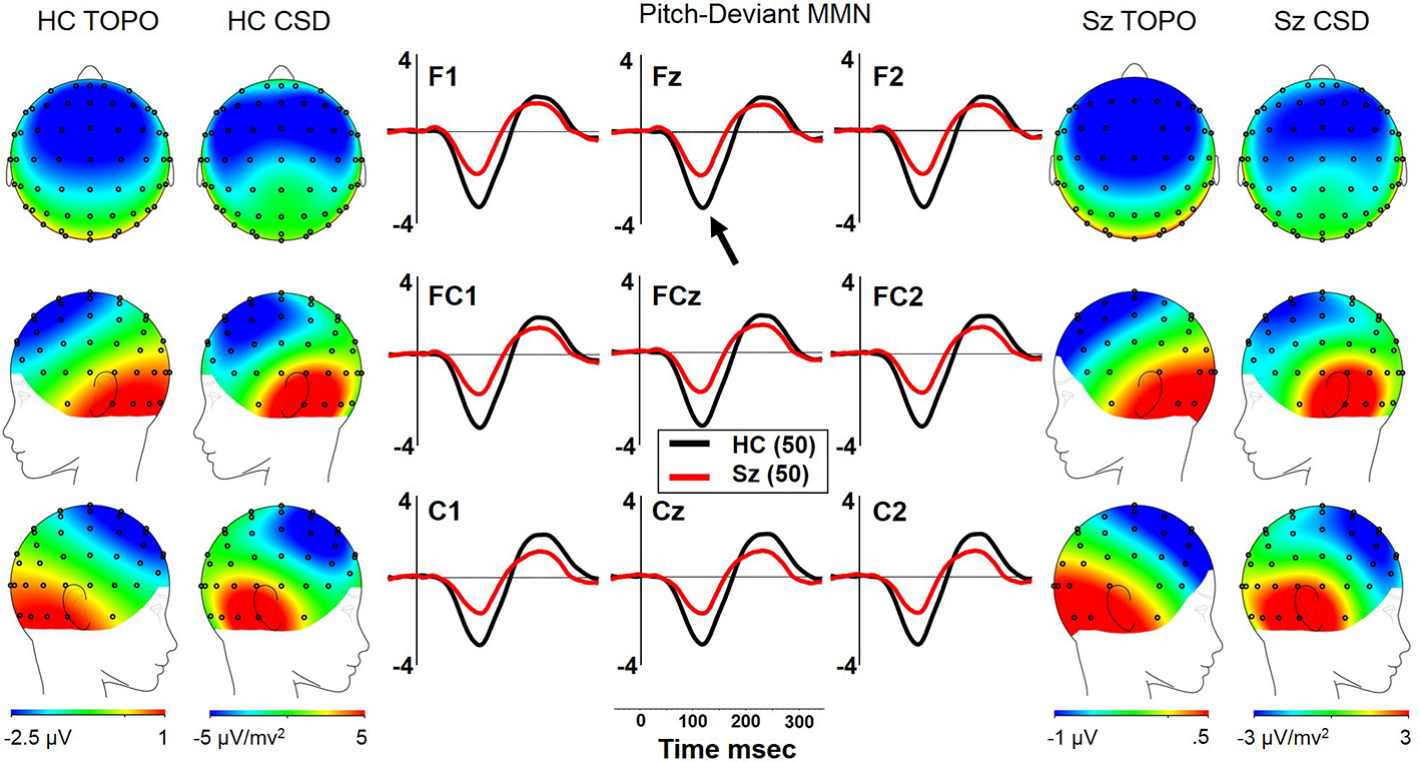
pMMN in long-term schizophrenia. HC, Healthy Control subjects; Sz, Long-term Schizophrenia subjects; Topo, Voltage topography; CSD, Current Source Density topography.

Both Sz and HCSz generated pMMN, and CSD analysis revealed the expected source-sink configuration consistent with an auditory cortex dipole generator (**Figure 1**). ANOVA revealed significant reductions of pMMN in Sz [F(1,98) = 12.4, p =.001; see **Table 3** for MMN values]. pMMN differed between electrode chains [F(2,196) = 29.3, p < .001, ϵ = 0.63] similarly in both groups, with larger MMN along the F and FC chains than the C chain. pMMN was also larger at the midline that the lateral sites [F(2,196) = 13.8, p < .001, ϵ = 0.85], similarly in both groups. Finally, the larger midline pMMN was more pronounced (more peaked) at the FC chain relative to F and C chains [F(4,392) = 6.8, p < .001, ϵ = 0.91], similarly in both groups.

**Table 3:** MMN values.

	F1	FZ	F2	FC1	FCz	FC2	C1	Cz	C2
pMMN									
**SZ**	−1.8 (1.6)	−1.9 (1.6)	−1.9 (1.6)	−1.8 (1.6)	−2.0 (1.7)	−1.9 (1.7)	−1.6 (1.6)	−1.7 (1.8)	−1.6 (1.7)
**HCSZ**	−3.0 (1.7)	−3.1 (1.7)	−3.1 (1.6)	−3.1 (1.8)	−3.3 (1.8)	−3.1 (1.8)	−2.6 (1.7)	−2.9 (1.7)	−2.6 (1.6)
									
**FESz**	−2.7 (1.8)	−2.7 (1.8)	−2.6 (1.7)	−2.7 (1.9)	−2.8 (1.9)	−2.6 (1.8)	−2.4 (1.9)	−2.4 (1.8)	−2.3 (1.7)
**HCFE**	−3.1 (1.6)	−3.1 (1.6)	−3.2 (1.6)	−3.2 (1.6)	−3.3 (1.7)	−3.2 (1.8)	−2.6 (1.7)	−2.7 (1.7)	−2.6 (1.6)
									
**dMMN**									
**SZ**	−0.9 (1.1)	−1.0 (1.2)	−1.0 (1.2)	−0.9 (1.2)	−1.2 (1.3)	−1.1 (1.3)	−0.8 (1.3)	−1.0 (1.3)	−1.0 (1.3)
**HCSZ**	−1.7 (1.6)	−1.8 (1.6)	−1.8 (1.6)	−1.9 (1.7)	−2.1 (1.9)	−1.9 (1.7)	−1.6 (1.7)	−1.7 (1.7)	−1.6 (1.5)
									
**FESz**	−2.1 (1.6)	−2.2 (1.7)	−2.2 (1.7)	−2.2 (1.6)	−2.4 (1.7)	−2.3 (1.7)	−1.9 (1.5)	−2.0 (1.6)	−2.0 (1.6)
**HCFE**	−2.2 (1.6)	−2.3 (1.5)	−2.4 (1.5)	−2.3 (1.6)	−2.5 (1.6)	−2.4 (1.6)	−1.9 (1.7)	−2.0 (1.6)	−2.0 (1.5)

Values are mean (SD). Voltage in μV, individual peak MMN ±25 ms.

#### pMMN Correlations With Clinical, Neuropsychological, and Social Functioning Measures

In Sz, larger pMMN was associated with worse PANSS positive factor scores (ρ = −.35, p = 0.012) and SAPS global scores (ρ = −.36, p = 0.011). PANSS total and negative factor scores, and SANS global scores did not correlate with pMMN. Larger pMMN in Sz was associated with higher WASI IQ (ρ = −.39, p = 0.005). On the MATRICS, larger pMMN was associated at trend-level with better Overall Composite scores (ρ = −.27, p = 0.063), and significantly with better Working Memory (ρ = −.30, p = 0.035), Reasoning and Problem Solving (ρ = −.34, p = 0.015), and Visual Learning (ρ = −.32, p = 0.024). Finally, in Sz, larger pMMN was associated with better UPSA-B finance scores (ρ = −.30, p = 0.035), but with no other social functioning measures. In matched HCSz, larger pMMN was associated on the MATRICS marginally with better Overall Composite scores (ρ = −.26, p = 0.065) and significantly with Visual Learning (ρ = −.30, p = 0.037).

#### dMMN

**Figure 2:**
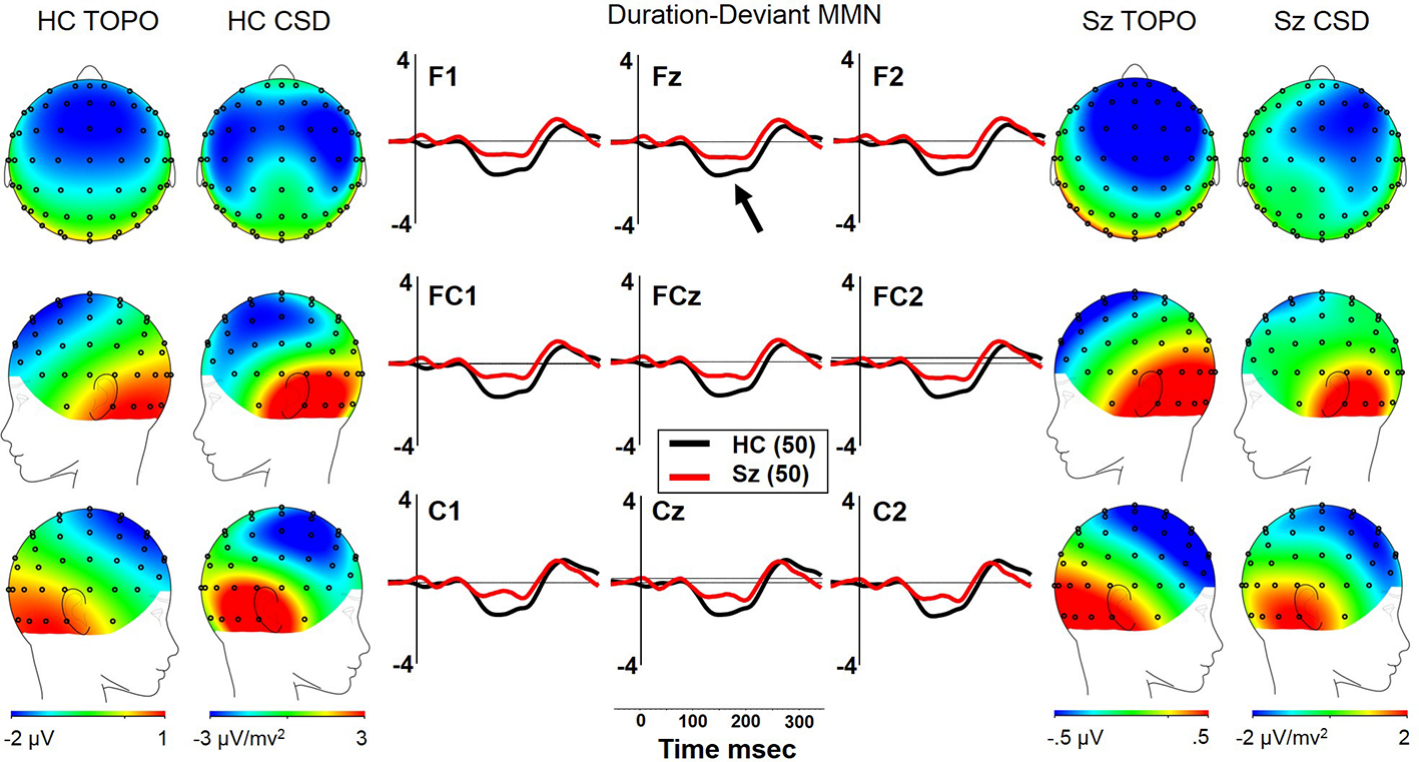
dMMN in long-term schizophrenia. HC, Healthy Control subjects; Sz, Long-term Schizophrenia subjects; Topo, Voltage topography; CSD, Current Source Density topography.

Both Sz and HCSz generated dMMN, and CSD analysis revealed the expected source-sink configuration consistent with an auditory cortex dipole generator (**Figure 2**). ANOVA revealed significant reductions of dMMN in Sz [F(1, 98) = 7.7, p =.006; see **Table 3** for MMN values]. dMMN differed between electrode chains [F(2, 196) = 14.0, p < .001, ϵ = 0.79] similarly in both groups, with largest dMMN along the FC chain, followed by the F chain then the C chain. dMMN was also larger at the midline than the lateral sites [F(2, 196) =15.0, p < .001, ϵ = 0.90], but laterality differed between groups [F(2, 196) = 3.9, p =.026, ϵ = 0.90]. Sz were somewhat right lateralized, whereas HC were more symmetrical (see **Figure 2** topography and CSD maps). Finally, dMMN differed significantly in topography between chains [F(4, 392) = 4.8, p =.006, ϵ = 0.59], with relatively greater midline than lateral (more peaked) dMMN at the FC chain relative to F and C chains, similarly in both groups.

#### dMMN Correlations With Clinical, Neuropsychological, and Social Functioning Measures

There were no significant correlations between dMMN and clinical symptoms in Sz. Larger dMMN was associated with higher WASI IQ (ρ = −.29, p = 0.042). On the MATRICS, larger dMMN was associated with better Visual Learning (ρ = −.28, p = 0.048). Finally, in Sz, larger dMMN was associated with worse interpersonal communication/interaction on the SFS (ρ =.29, p = 0.049). There were no other significant associations between dMMN and social functioning scores. In matched HCSz, no significant correlations between dMMN and neuropsychological measures were observed.

### First Episode Sz-Spectrum

#### pMMN

**Figure 3:**
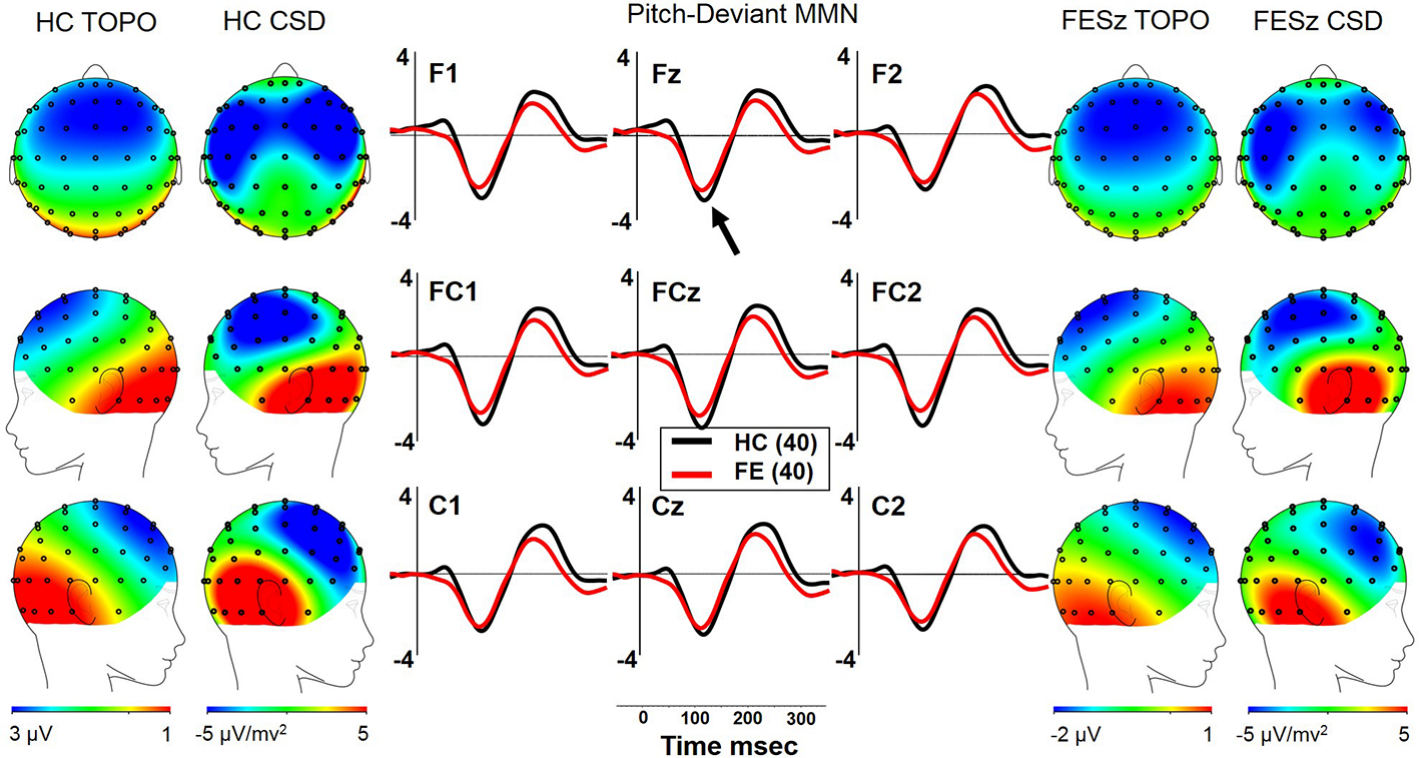
pMMN in the first episode schizophrenia spectrum. HC, Healthy Control subjects; FESz, First Episode Schizophrenia Spectrum subjects; Topo, Voltage topography; CSD, Current Source Density topography.

Both FESz and HCFE generated pMMN, and CSD analysis revealed the expected source-sink configuration consistent with an auditory cortex dipole generator (**Figure 3**). ANOVA revealed no significant reductions of pMMN in FESz [F(1, 78) = 1.2, p =.27; see **Table 3** for MMN values]. pMMN differed between electrode chains [F(2, 156) = 39.5, p < .001, ϵ = 0.67] with larger MMN along the F and FC chains than the C chain. However, there was a trend-level difference in amplitude across chains between groups [F(2, 156) = 3.0, p =.07, ϵ = 0.67], with HC showing a more marked drop-off along the C chain. pMMN differed significantly in topography between chains [F(4, 312) = 3.2, p =.029, ϵ = 0.66], with relatively greater midline than lateral (more peaked) pMMN at the FC chain relative to F and C chains, similarly in both groups.

#### pMMN Correlations With Clinical, Neuropsychological, and Social Functioning Measures

**Figure 4:**
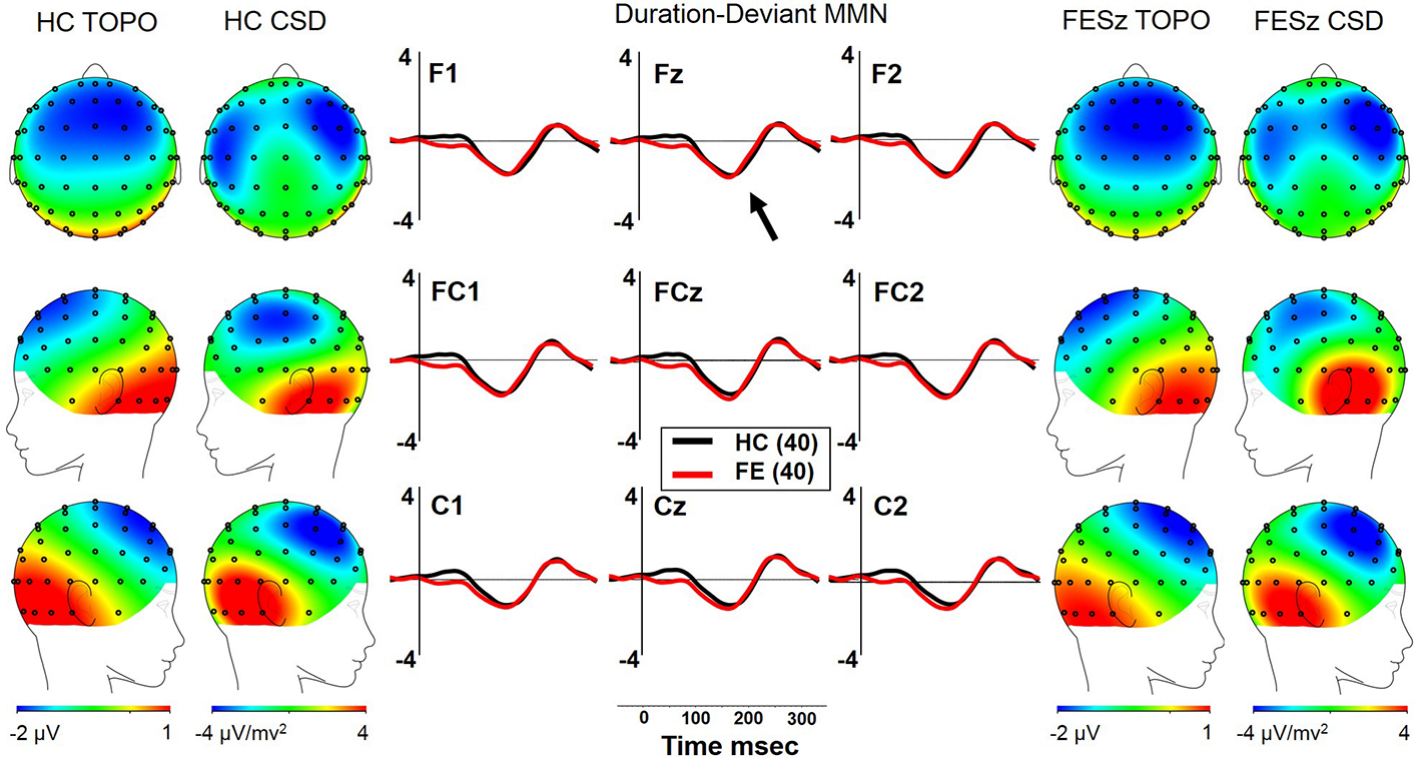
dMMN in the first episode schizophrenia spectrum. HC, Healthy Control subjects; FESz, First Episode Schizophrenia Spectrum subjects; Topo, Voltage topography; CSD, Current Source Density topography.

In FESz, larger pMMN was associated with lower PANSS Negative factor scores (ρ =.45, p = 0.003). PANSS Total and Positive factor scores, and SANS and SAPS global scores did not correlate with pMMN in FESz. On the MATRICS, larger pMMN was associated marginally with better Overall Composite scores (ρ = −.31, p = 0.056) and significantly with better Reasoning and Problem Solving (ρ = −.33, p = 0.036). Finally, in FESz, pMMN was associated with several measures of social functioning (**Figure 4**). Larger pMMN was associated with better GAS scores (ρ = −.32, p = 0.048) and UPSA-B finance scores (ρ = −.33, p = 0.047). On the GF, larger pMMN in FESz was associated marginally with improved current social functioning (ρ = −.30, p = 0.064) and significantly with better current role functioning (ρ = −.32, p = 0.045). Of primary importance for progressive dysfunction prior to the emergence of psychosis, healthier pMMN was significantly associated with highest role functioning in the year prior to first episode (ρ = −.39, p = 0.014). No correlations in HCFE with pMMN were significant.

#### dMMN

**Figure 5:**
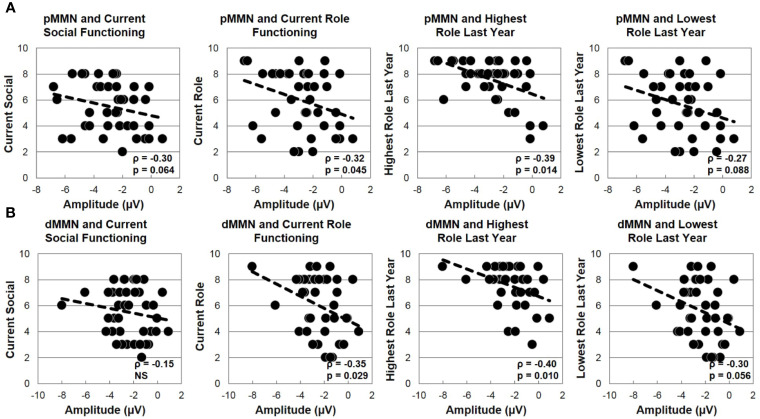
MMN and social functioning in the first episode schizophrenia spectrum. **(A)** Correlations with pMMN. **(B)** Correlations with dMMN.

Both FESz and HCFE generated dMMN, and CSD analysis revealed the expected source-sink configuration consistent with an auditory cortex dipole generator (**Figure 5**). ANOVA revealed no significant difference between groups in dMMN [F(1, 78) = 0.4, p =.84; see **Table 3** for MMN values]. dMMN differed between electrode chains [F(2, 156) = 24.1, p < .001, ϵ = 0.65] similarly in both groups, with larger dMMN along the F and FC chains than the C chain. dMMN was also smaller at the left sites relative to the middle and right sites [F(2, 156) = 7.3, p =.002, ϵ = 0.80], similarly in both groups (see topography and CSD maps in **Figure 5**). No other main effects or interactions were significant.

#### dMMN Correlations With Clinical, Neuropsychological, and Social Functioning Measures

In FESz, larger dMMN was marginally correlated with lower PANSS Negative factor scores (ρ =.30, p = 0.063), but not with PANSS Total or Positive factor scores, or SANS or SAPS scores. Larger dMMN was associated with higher WASI IQ (ρ = −.36, p = 0.022). On the MATRICS, larger dMMN was associated marginally with better Overall Composite scores (ρ = −.30, p = 0.060) and Reasoning and Problem Solving (ρ = −.31, p = 0.054), and significantly with better Speed of Processing (ρ = −.31, p = 0.050) and Visual Learning (ρ = −.31, p = 0.048). Finally, in FESz, dMMN was associated with several measures of social functioning (**Figure 4**). Larger dMMN was associated with better UPSA-B finance scores at trend-level (ρ = −.30, p = 0.069). Larger dMMN was marginally associated with better SFS independence and performance (ρ = −.31, p = 0.051). On the GF, better dMMN in FESz was associated with better current social role functioning (ρ = −.35, p = 0.029). Of primary importance for progressive dysfunction prior to transition to psychosis, as for pMMN, healthier dMMN was significantly associated with the highest role functioning in the year prior to first episode (ρ = −.40, p = 0.010), and marginally with the lowest role functioning in the last year (ρ = −.30, p = 0.056). In matched HCFE, no correlations with dMMN were significant.

## Discussion

In Sz, pMMN and dMMN were reduced. Frontal topography of pMMN did not differ between groups, while dMMN showed a right-ward asymmetry in Sz. Paradoxically, larger pMMN was associated with more positive symptoms, whereas dMMN was not correlated with clinical symptoms. Although the association between greater positive symptoms and less pMMN deficit may indicate more normal deviance detection in more positive symptoms, the meta-analysis of Erickson et al. (10) suggests no reliable association across studies. Healthier pMMN and dMMN in Sz were associated with better overall cognitive functioning, but in this large sample were not associated with better social functioning or social cognition, with the exception of better UPSA-B finance for pMMN, but worse interpersonal communication for dMMN. Finding reduced MMN moderately associated with impaired intellect is generally consistent with the literature. However, a strong link between dMMN and social functioning was absent in long-term Sz.

In FESz, pMMN and dMMN were not reduced relative to matched psychiatrically-well comparison subjects. While the FESz group mean was not reduced, several pathological correlations were observed between MMN measures and clinical, neuropsychological, and social behavior. Higher PANSS negative factor scores were associated with smaller pMMN and dMMN amplitudes, but this association was not present for the SANS. Recall in Sz, no associations with negative symptoms were observed, and increased positive symptoms were associated with larger pMMN. In our previous report of an independent sample of FESz, lower pMMN and dMMN amplitudes were associated with increased positive symptoms (9). Although the association between MMN and negative symptoms (and social functioning) is consistent with the recent SEM analysis (33), given the variability in the findings and the recent meta-analysis suggesting no consistent link between MMN and symptoms (10), we suggest caution be used in interpreting this symptom correlation.

Smaller pMMN and dMMN were associated with impaired cognitive functioning in Sz and FESz. This replicates our initial report of such an association in FESz (9), but, unlike our previous study, in this larger independent sample, a similar association was not present for HC, except for a weak association with MATRICS subtests. These data suggest that intellectual functioning in FESz is impacted by the pre-psychosis pathological process giving rise to the prodromal progression of impairment to psychosis. With our previous demonstration of pathological associations between pMMN and gray matter volumes at first psychosis and correlated changes in left auditory cortex gray matter volume and pMMN post-onset (14), and other reports of progressive cortical loss during the prodrome (27, 28), we speculate that MMN may serve as a robust link between progressive prodromal gray matter pathology and impaired executive functioning. However, definitive longitudinal studies in clinical high risk individuals are needed.

Novel to this report is the finding that pMMN and dMMN at first psychosis (on average 17.2 days after first clinical contact) for psychosis, are not only associated with impaired intellectual functioning, but with current and prodromal social functioning. The most robust associations were with the highest level of role performance in the last year, during the pre-psychosis prodromal stage. This represents the first report of an association between MMN and current and prodromal social functioning in FESz, and indicates that pMMN and dMMN are also sensitive indices of performance prior to psychosis. Further research is needed to clarify the association between social functioning and MMN at first episode Sz and during the prodrome, and whether such relationships are mediated by gray matter loss, cognitive impairment, or sub-clinical symptom emergence.

Several caveats should be mentioned. Our correlations were not adjusted for multiple comparisons. In that light they must be considered exploratory, even in the light of *a priori* hypotheses of directional associations. Thus, the reader is urged to interpret findings cautiously, and results are in need of replication. Previous findings show an association between social functioning and dMMN amplitude in Sz (32, 33). We did not find strong associations between social functioning and MMN amplitude in the older Sz group. It is not known why our Sz sample did not demonstrate such associations. UPSA-B finance showed associations in both SZ and FESz, yet the task is somewhat outdated, so the relevance for real-world function may be questioned. Secondly, some participants received a shortened version of the MMN protocol in which only 800, as opposed to 1600, tones were presented. Signal to noise differences might affect the MMN measurements. However, follow up analyses showed no significant difference in the first 800 trials MMNs compared to the second 800 trials MMNs. Thirdly, participants were ruled out for drug use, with the exception of marijuana and alcohol. While we did screen for drug use and perform urinalysis for current drug use, we did not collect detailed measures of drug use appropriate for covariate analysis. Finally, both SZ and FESz had relatively high estimates of premorbid intellect (WASI), and were matched to respective HC on WASI scores. However, both SZ and FESz were significantly reduced on the MATRICS overall scores, tests selected to be sensitive to the effects of psychosis on cognition. Thus, these samples are certainly not free from the effects of illness of cognitive function.

Our findings show that while neither pMMN nor dMMN are reduced at the first clinical contact for psychosis, both pMMN and dMMN are indices of current intellectual and social impairments in FESz. Of primary importance is the novel finding that pMMN and dMMN at first break also correlate with social functioning in the pre-psychosis prodromal stage of Sz. This finding is consistent with the presence of progressive neural impairment prior to the onset of psychosis reflected in a cascade of progressive disability, particularly in social functioning. Further, pMMN and dMMN appear to be sensitive to this progressive prodromal course, making them potentially powerful tools to understand the underlying mechanisms of progressive structural and functional impairments prior to and subsequent to psychosis. Current work is testing MMN, gray matter volumes, and social and cognitive functioning in clinical high risk individuals.

## Data Availability Statement

The datasets generated for this study are available on request to the corresponding author.

## Ethics Statement

The studies involving human participants were reviewed and approved by University of Pittsburgh IRB. The patients/participants provided their written informed consent to participate in this study.

## Author Contributions

TM provided substantial contributions to the design, acquisition, analysis, and interpretation of data for the work, and drafting the work and revising it critically for important intellectual content. SH provided substantial contributions to the acquisition, analysis, and interpretation of data for the work, and revising the report critically for important intellectual content. BC provided substantial contributions to the acquisition, analysis, and interpretation of data for the work, and revising the report critically for important intellectual content. DS provided substantial contributions to the conception and design of the work; the acquisition, analysis, and interpretation of data for the work; and drafting the work and revising it critically for important intellectual content. All authors provided approval for publication of the content, and agree to be accountable for all aspects of the work in ensuring that questions related to the accuracy or integrity of any part of the work are appropriately investigated and resolved.

## Funding

This work was supported by NIH R01 MH094328 to DS.

## Conflict of Interest

The authors declare that the research was conducted in the absence of any commercial or financial relationships that could be construed as a potential conflict of interest.
